# Testing the Duke Population Health Profile (Duke-PH) in a Sample of Community Health Center Patients

**DOI:** 10.3389/fpubh.2019.00248

**Published:** 2019-09-04

**Authors:** George R. Parkerson, Howard J. Eisenson, Colin Campbell

**Affiliations:** ^1^Department of Community and Family Medicine, Duke University School of Medicine, Durham, NC, United States; ^2^Lincoln Community Health Center, Durham, NC, United States; ^3^Department of Sociology, East Carolina University, Greenville, SC, United States

**Keywords:** population health, determinants of health, health-related quality of life, health status, patient-reported outcomes, patient surveys, underserved populations

## Abstract

**Introduction:** Our purpose was to develop and test a brief, self-report, and scorable survey instrument for measuring population health profiles from the individual respondent's perspective. We defined population health as the state of physical, mental, and social well-being of a group of individuals, including determinants of their well-being.

**Materials and Methods:** Respondents were adult patients in a community health center. Instrument items, an overall scale, and two subscales were developed and evaluated. Reliability was tested by Cronbach's alphas and test-retest correlations; construct validity was tested by correlations between scores and economic and clinical factors; criterion validity was tested by regression analyses for prediction of morbidity and health care utilization by baseline scores; and feasibility was tested by length of administration time.

**Results:** This was a 2-years prospective study of 450 patients, mostly black non-Hispanics (54%) and Hispanics (29%), many with no health insurance (45%), and poor enough to meet the federal poverty level (73%). The Duke Population Health Profile (Duke-PH) was developed with a 14-item PH scale for overall population health profile and two 7-item subscales, one for social determinants and the other for health determinants. Validity of item selection was indicated by item convergent and item discriminant correlations. Scale and subscale reliability were supported for internal consistency by Cronbach's alphas of 0.63–0.73, and for temporal stability by test-retest correlations of 0.65–0.78. Support for construct validity was shown by the more favorable baseline subscale and scale mean scores for patients able to buy private insurance than for patients unable to afford it. Criterion validity was supported by regression analyses showing that baseline scale and subscale scores predicted both baseline morbidity and 6-months utilization. Feasibility was shown by the mean self-administration time of 3.9 min and mean interviewer-administration time of 5.8 min.

**Discussion:** The strength of this study is support for Duke-PH reliability, validity, and feasibility in a community health center patient population. The new instrument is unique because it measures both social and health determinants of population health from the perspective of individuals in the population.

## Introduction

Our purpose was to design a brief, self-report, and scorable survey instrument for measuring population health profiles from the individual respondent's perspective and to test the instrument for reliability, validity, and feasibility. In the aggregate, these individual profiles can offer information about the population health of a group of individuals. Although the concept of population health is key to efforts for making health care more effective, there is no consensus on a single definition (1–3). Definitions have been very broad, as illustrated by Kindig and Stoddart's definition, “health outcomes, patterns of health determinants, and policies and interventions that link these two (4).” They listed determinants of population health as “medical care, public health interventions, aspects of the social environment (income, education, employment, social support, culture) and of the physical environment (urban design, clean air and water), genetics, and individual behavior (4).”

In 1948, individual health was defined by the World Health Organization (WHO) as “…a state of complete physical, mental, and social well-being and not merely the absence of disease or infirmity (5).” In 2008, because of increased emphasis on social determinants, the WHO Commission on Social Determinants of Health stated, “Together, the structural determinants and conditions of daily life constitute the social determinants of health and are responsible for a major part of health inequities between and within countries (6).”

Because both the Kindig/Stoddart definition of population health and WHO definitions of individual health are important, we combined them when developing the Duke Population Health Profile (Duke-PH), a metric that measures population health profiles and evaluates population health as “the state of physical, mental, and social well-being of a group of individuals, including determinants of their well-being.” This definition is compatible with the 2013 Institute of Medicine (IOM) report on population health measures (7), which summarizes the Healthy People 2020 foundation health measures (8) in four domains: general health status, health-related quality of life and well-being, determinants of health, and disparities.

We realized that our new self-report instrument would necessarily be limited to measurement of aspects of well-being that respondents could perceive personally, such as their personal social determinants, and their personal health determinants. Current self-report health instruments, such as the Short Form 12 (SF-12) (9) and the Duke Health Profile (DUKE) (10), measure individual physical, mental, and social health by the WHO definition. However, they concentrate on physical and mental health and illness, without measuring many of the social determinants listed by Kindig and Stoddart. Some instruments, such as the Duke Social Support and Stress Scale (DUSOCS) (11), measure certain social determinants, but do not include many of the other determinants now recognized as important.

Examples of recent self-report population health instruments are HealthBegins with 27 items (12) and PRAPARE with 21 items (13) both of which measure mostly social determinants. Of the two, only HealthBegins is designed in such a way that it can be scored. Scoring is important to identify individuals and groups at risk for poor health outcomes, to facilitate planning and evaluation of interventions for improving outcomes and reducing costs, and to compare health status among different populations.

## Materials and Methods

### Data Collection

This was a prospective study of primary care patients. After verbal consent, patients completed the test instrument themselves, or had it administered by the interviewer if they had poor eyesight or low literacy. For patients who spoke only Spanish, translations were used with the help of an interpreter. During the first part of the study, enrollees were contacted by the interviewer 1 month later for follow-up administration over the phone. Diagnostic and utilization data were collected from the Duke Medical Center electronic health record (Duke EHR). Data verification was performed on a 10% patient sample by an independent auditor.

### Development of Instrument Items and Scales

Items for the instrument were selected by the investigators, based upon their past experience with instrument development and patient care. Five of the 14 items came directly from the DUKE (10), a scale well-validated since its inception in 1990. The scale and two subscales were selected and tested using the two phases suggested by Boateng et al. (14), i.e., “scale development,” and “scale evaluation.” Scale and subscale development included item-remainder correlations between single item scores and their own scale or subscale scores that did not include their own item score (item convergent validity), and between single item scores and the scores of the opposite subscale (item discriminant validity). Evaluation consisted of reliability testing for internal consistency and temporal stability. Validity testing included construct validity and criterion predictive validity.

### Statistical Methods

Item convergent and item discriminant validity were tested by Spearman rank-order correlations. Reliability was tested by Cronbach's alpha (15) for internal consistency of item scores within their scales and subscales, and by test-retest Spearman correlations for score consistency over time. A re-test period of 4 weeks was chosen arbitrarily, while recognizing that multiple factors in a medical setting can change over any given time period. Construct validity was tested by associating scale and subscale scores with related socioeconomic and clinical factors using analysis of variance (ANOVA) and Spearman correlations. Criterion validity was measured by demonstrating the predictive effect of baseline scale and subscale scores for morbidity (number of individual patient health problems at time of the baseline encounter) and for health care utilization (number of 6-months return encounters after the baseline encounter) using multiple regression analyses that controlled for the following 11 health-related factors: age, gender, race/ethnicity, marital status, number of people in the household, health insurance status, BMI, presence of hypertension, presence of diabetes mellitus, current alcohol use, and current cigarette smoking. All types of health problems and encounters documented in the Duke EHR were included.

### Feasibility

Feasibility was assessed by the time required for administration of the instrument.

### Ethics Approval

All procedures performed in this study involving human participants were in accordance with the ethical standards of the 1964 Helsinki declaration and its later amendments and of the Duke University Health System Institutional Review Board (IRB). The IRB approved the study under the protocol number Pro00069809. Informed consent was obtained from all individual participants included in the study. Because the study was a minimum risk study, the IRB approved a waiver of written consent, but required reading of an IRB-approved consent form to participants by the interviewer, followed by verbal consent if they agreed to be in the study.

## Results

### Patient Characteristics

Respondents were a convenience sample of 450 adult patients at the Lincoln Community Health Center (Lincoln), a Federally-Qualified Health Center serving approximately 33,000 patients, mostly in Durham, North Carolina. Lincoln patient data are entered routinely into the Duke EHR system.

As shown in Table 1, patients had a wide range of ages and higher percentages of females, singles, black non-Hispanics, and those with no health insurance. Not shown in the table, annual household income (reported by 275 patients) varied from $121 to $121,448 (median $13,284). Income was low enough to meet the federal poverty level for 73% of those patients. Fifty-seven percent had hypertension, 33% had diabetes mellitus, 26% had both hypertension and diabetes, 19% were current alcohol drinkers, and 18% were current cigarette smokers. The median body mass index (BMI) was 31.5 kg/m^2^, indicating that over half were obese, i.e., BMI >30 kg/m^2^. A total of 3,987 health problems were recorded for the 450 patients (8.9 per patient), using the ICD-10 classification (16).

**Table 1:** Socioeconomic characteristics.

**Continuous variables**	**Mean**	**SD**
Age	51.4	14
People in household	2.4	1.7
**Categorical variables**	**Frequency**	**Percent**
Gender			
Female	275	61
Male	175	39
Race/ethnicity
Black (non-Hispanic)	241	54
Hispanic	129	29
White (non-Hispanic)	62	14
Other	18	4
Marital status			
Never married	201	45
Married	126	28
Divorced	51	11
Widowed	30	7
Separated	31	7
Not reported	11	2
Insurance status			
None	201	45
Medicaid only	52	12
Medicare only	89	20
Medicaid and Medicare	28	6
Private	65	14
Other	14	3

*n = 450*.

During the 6 months following administration of the instrument, 418 (93%) of the study patients had at least one encounter at Lincoln or Duke University Health System clinical facilities. There were 4,725 total encounters (10.5 per patient), of which 33% were telephone or e-mail contacts, 18% office visits with primary care providers, 16% contacts with non-provider personnel, 11% office visits with specialty providers, 7% non-surgical procedures, 5% hospital inpatient days, 5% emergency department visits, 3% miscellaneous encounters, and 2% surgical and anesthesia procedures.

### Item Development

We designed the new 14-item Duke Population Health Profile (Duke-PH) instrument, which is shown in full on the website of the Duke Department of Family Medicine and Community Health (FMCH) (17). Five items came from the DUKE (10) to measure perceived health, pain, depressed feelings, social interactions, and health confinement. The other nine items were newly created to measure illness burden, health care quality, money for basic needs, living conditions, support vs. stress, education, employment, discrimination, and health care visits.

### Scale and Subscale Development

The Population Health Profile Scale (PH scale) consisted of all 14 items. This scale was divided into two subscales: (1) Social Determinants Subscale (social subscale), with seven items: money for basic needs, living conditions, support vs. stress, education, employment, discrimination, and social interactions, and (2) Health Determinants Subscale (health subscale), with seven items: perceived health, illness burden, health care quality, pain, depressed feelings, health care visits, and health confinement. Scores ranged from 0 for the most unfavorable level of health, to 100 for the most favorable. The only possible item scores are 0, 50, and 100 because there are only three response options for each item. However, a range of scores from 0 to 100 are possible for scale and subscale scores because they represent the mean of groups of item scores. The complete scoring system is shown on the FMCH website (17).

As shown in Table 2, mean item scores ranged from the unfavorable score of 40.2 for “money for basic needs” to the favorable score of 86.4 for “health care quality.” The floor value for “money for basic needs” indicates that 42% of patients reported they did not have enough money (score = 0), and the ceiling value indicates that only 23% of patients reported having enough money (score = 100). Other items with unfavorable scores included “pain,” i.e., having much pain (score = 46.6), and “perceived health,” i.e., not feeling healthy (55.9). Other items with favorable scores were “discrimination,” i.e., not feeling discriminated against (82.0), and “living conditions,” i.e., good living conditions (78.9). The mean subscale scores of 68.8 for the social subscale and 68.4 for the health subscale indicated these two different types of determinants had very similar ratings in this study population. Other analyses showed the correlation of 0.44 (*p* < 0.001) between the social and health subscale scores.

**Table 2:** Distribution of duke-PH scale, subscale, and item scores.

**Duke-PH scale, subscales, and items**	Mean ± SD^*^	**Minimum**	**Maximum**	**Median**	**Floor %^†^**	**Ceiling %^‡^**
Population health scale (All 14 items)	68.6 ± 15.9	21.4	100	67.9	<1	2
Social determinants subscale (7 items)	68.8 ± 19.1	7.1	100	71.4	<1	6
Money for basic needs	40.2 ± 39.1	0	100	50	42	23
Living conditions	78.9 ± 32.0	0	100	100	8	66
Support vs. stress	69.3 ± 36.2	0	100	100	14	53
Education	65.1 ± 36.6	0	100	50	16	46
Employment	79.9 ± 34.4	0	100	100	12	71
Discrimination	82.0 ± 29.8	0	100	100	6	70
Social interactions	66.3 ± 31.9	0	100	50	9	42
Health determinants subscale (7 items)	68.4 ± 18.4	7.1	100	71.4	<1	4
Perceived health	55.9 ± 31.7	0	100	50	15	27
Illness burden	56.9 ± 40.2	0	100	50	26	40
Health care quality	86.4 ± 25.7	0	100	100	3	76
Pain	46.6 ± 34.9	0	100	50	28	21
Depressed feelings	69.0 ± 34.8	0	100	100	12	50
Health care visits	79.8 ± 30.4	0	100	100	6	66
Health confinement	84.2 ± 31.4	0	100	100	9	77

*n = 450*.

*0, most unfavorable score; 100, most favorable score*.

* *SD, standard deviation*.

† *Floor %, percent of patients with the minimum (most unfavorable) score*.

‡ *Ceiling %, percent of patients with the maximum (most favorable) score*.

Item convergent validity for the PH scale is shown in Table 3. All 14 items had statistically significant (*p* < 0.001) correlations with the scale score after removal of the index item score, ranging from 0.19 for “health care visits” to 0.47 for “illness burden.” Item convergent validity for the two subscales is shown in Table 4, where each item score had statistically significant correlations (*p* < 0.001) with its own subscale score after removal of its own item score, ranging from 0.27 to 0.50 for social items and from 0.23 to 0.47 for health items. Also in Table 4, item discriminant validity was supported for five of the seven social items and five of the seven health items. For example, the correlation of the item score for “living conditions” with its own social subscale score (0.50) was higher than its correlation with the health subscale score (0.24). Even though two of the other social items, “money for basic needs” and “support vs. stress,” had high correlations with their own social subscale score (0.31 and 0.36, respectively), their correlations with the health subscale score were slightly higher (0.34 and 0.39, respectively), thereby demonstrating convergent, but not discriminant validity for these two items. Likewise, the item scores for two of the seven health subscale items, “illness burden” and “depressed feelings,” correlated somewhat higher with the social subscale score (0.40 and 0.40, respectively) than with their own health subscale score (0.37 and 0.34, respectively).

**Table 3:** Convergent validity of item selection for the Duke-PH scale.

**Items**	**Item to scale correlations^†^**
Money for basic needs	0.38^*^
Living conditions	0.42^*^
Support vs. stress	0.45^*^
Education	0.31^*^
Employment	0.24^*^
Discrimination	0.33^*^
Social interactions	0.29^*^
Perceived health	0.35^*^
Illness burden	0.47^*^
Health care quality	0.23^*^
Pain	0.38^*^
Depressed feelings	0.43^*^
Health care visits	0.19^*^
Health confinement	0.33^*^

*n = 450*.

† *Item remainder Spearman rank-order correlations between item and scale scores, after removing the index item score from the scale score (convergent validity)*.

* *p <0.001*.

**Table 4:** Convergent and discriminant validity of item selection for the Duke-PH subscales.

**Items**	**Social determinants subscale^†^**	**Health determinants subscale^†^**
Social determinants subscale items		
Money for basic needs	0.31^**^	0.34^**^
Living conditions	0.50^**^	0.24^**^
Support versus stress	0.36^**^	0.39^**^
Education	0.34^**^	0.19^**^
Employment	0.29^**^	0.11^*^
Discrimination	0.34^**^	0.23^**^
Social interactions	0.27^**^	0.25^**^
Health determinants subscale items		
Perceived health	0.25^**^	0.36^**^
Illness burden	0.40^**^	0.37^**^
Health care quality	0.16^**^	0.23^**^
Pain	0.20^**^	0.47^**^
Depressed feelings	0.40^**^	0.34^**^
Health care visits	0.06^NS^	0.27^**^
Health confinement	0.19^**^	0.40^**^

*n = 450*.

† *Spearman rank-order correlations between item scores and their own subscale scores (shown in bold font) are item remainder correlations that do not include the index item score in the subscale score (for indication of convergent validity). Correlations between item scores and the other subscale scores (shown in regular font) include all item scores in the subscale scores (for indication of discriminant validity when compared with scores in bold font)*.

* p <0.05,

** *p <0.001, NS = Not statistically significant*.

### Scale and Subscale Evaluation

Scale and subscale reliability in terms of internal consistency of item scores was supported by Cronbach's alphas at the time of initial face-to-face administration for all 450 patients and again 1 month later for telephone administration for a sample of 203. For the initial group, alphas were 0.73 for the 14-item PH scale, 0.63 for the 7-item social subscale, and 0.63 for the 7-item health subscale. For the telephone sample, alphas were 0.75, 0.62, and 0.64, respectively. The acceptable Cronbach's alpha varies among experts in the field, e.g., >0.50 by Helmstadter (18) and >0.70 by Nunnally (19). Lower alphas are expected for scales with fewer items and wider variation in item content (20). Reliability in terms of temporal stability was supported by 1-month test-retest correlations of 0.78 for PH scale scores, 0.75 for social subscale scores, 0.65 for health subscale scores, all with *p* < 0.0001. Correlations >0.80 are considered acceptable (21), but only for populations, unlike ours, in which no change is expected in causative factors between test and re-test times.

Construct validity was supported by ANOVA analyses of health insurance type as an economic factor, which showed that patients who were financially able to buy private health insurance had a more favorable social subscale score (76.2) than patients with other types of insurance (60.7–74.9), model *F* = 5.46, *p* < 0.0001, a more favorable health subscale score (75.9) than those with other types of insurance (60.2–69.6), model *F* = 4.46, *p* < 0.001, and a more favorable PH scale score (76.0) than those with other types of insurance (57.1–72.2), model *F* = 6.20, *p* < 0.0001. Also, the negative Spearman correlation of −0.11 (*p* < 0.05) between BMI levels and health subscale scores indicated that heavier patients had worse personal health. Correlations between BMI levels and social subscale and PH scale scores were not statistically significant.

Criterion validity was demonstrated by multiple linear regression analyses showing that the Duke-PH scale score and both the social and health subscale scores were statistically significant predictors of both morbidity and utilization. As shown in Table 5 by the negative coefficients for scale scores, less favorable Duke-PH scale and subscale scores predicted greater morbidity and higher utilization, while controlling for the 11 other health-related independent variables. Regression model statistics ranged from R-Square = 0.12, *F* = 4.6 (*p* < 0.0001) for the model in which social subscale scores predicted utilization, to R-Square = 0.34, *F* = 17.4 (*p* < 0.0001) for the model in which health subscale scores predicted morbidity. Other statistically significant predictors of greater morbidity were older age, female gender, having health insurance, higher BMI, having hypertension, and having diabetes. Other predictors of higher utilization were having health insurance and drinking alcohol.

**Table 5:** Duke-PH scores as predictors of number of initial health problems (morbidity) and number of 6-months follow-up encounters (utilization), while controlling for other variables^†^.

	**Initial health problems**			**Follow-up encounters**		
	**Coefficient (SE^‡^)**	**Coefficient (SE^‡^)**	**Coefficient (SE^‡^)**	**Coefficient (SE^‡^)**	**Coefficient (SE^‡^)**	**Coefficient (SE^‡^)**
Social determinants subscale score	−0.031^*^ (0.015)	-	-	−0.057^*^ (0.028)	-	-
Health determinants subscale score	-	−0.066^***^ (0.015)	-	-	−0.174^***^ (0.028)	-
Population health scale score	-	-	−0.066^***^ (0.017)	-	-	−0.156^***^ (0.033)
Age	0.095^***^ (0.025)	0.096^***^ (0.025)	0.094^***^ (0.025)	0.014 (0.048)	0.013 (0.046)	0.008 (0.047)
Female	1.135^*^ (0.566)	0.969 (0.554)	1.099^*^ (0.558)	1.367 (1.095)	0.997 (1.049)	1.327 (1.068)
Has health insurance	2.013^**^ (0.630)	2.035^**^ (0.617)	2.072^*^ (0.622)	3.869^**^ (1.219)	3.994^**^ (1.168)	4.054^**^ (1.192)
Body mass index (BMI)	0.118^**^ (0.036)	0.101^**^ (0.035)	0.108^**^ (0.035)	0.111 (0.069)	0.066 (0.067)	0.088 (0.068)
Hypertensive	1.644^*^* (0.674)	1.862^**^ (0.664)	1.77^**^ (0.667)	−0.556 (1.305)	0.045 (1.256)	−0.231 (1.278)
Diabetic	2.047^**^ (0.608)	1.935^**^ (0.597)	1.952^**^ (0.601)	1.850 (1.176)	1.501 (1.130)	1.589 (1.151)
Alcohol drinker	1.096 (0.710)	0.920 (0.698)	0.975 (0.702)	3.210^*^ (1.373)	2.701^*^ (1.320)	2.894^*^ (1.345)
Intercept	−0.718 (2.240)	1.969 (2.218)	1.933 (2.319)	8.132 (4.334)	17.028^***^ (4.196)	15.634^**^ (4.443)
*R*^2^	0.31	0.34	0.33	0.12	0.19	0.16
*F* statistic	15.5^***^	17.4^***^	16.8^***^	4.6^***^	7.9^***^	6.4^***^

*n = 424*.

†Multiple linear regressions. Not shown in table are four additional independent variables (marital status, race/ethnicity, household size, and cigarette smoker), none of which were statistically significant predictors in any of the models.

*‡SE, Standard error*.

*p <0.05,

**p <0.01,

***p <0.001.

### Feasibility

Feasibility was indicated by the average time of 3.9 min (range 1–12) for self-administration of the Duke-PH by 85% of patients, and 5.8 min (range 3–17) for interviewer-administration for the other 15%, most of whom spoke only Spanish and required an interpreter. Of all patients, 78% reported no difficulty answering the questions.

## Discussion

Validation of self-report health status instruments has become more complex over the past 30 years, as indicated by the methodology proposed in the 2010 COSMIN study (22). We chose to adapt the scale development and evaluation method published by Boateng et al. (14) in 2018. Our analyses provided support for feasibility and partial support for both reliability and validity of the new Duke-PH. Validity of item selection and distribution within the PH scale, social subscale, and health subscale were supported by item convergent correlations of item scores with their own scale and subscale scores. However, in the item discriminant correlations we found that the two subscales were not completely independent of each other, because four of their items correlated higher with scores of the opposite subscale than with scores of their own subscale. We decided to leave the non-independent items in their initially assigned subscales, because we recognized the obvious clinical crossover of social and health factors for items like “money for basic needs” and “depressed feelings.” This crossover is a reminder that both personal social determinants and personal health determinants should be included in self-reported measurement of population health.

The Cronbach's alphas for internal consistency reliability for the Duke-PH scale scores and both subscale scores met psychometric standards, while test-retest correlations for temporal stability were somewhat lower than standards. However, test-retest standards may not be appropriate in this clinical setting, where health-related factors can be expected to change during a 1-month re-test period, depending upon course and severity of disease, impact of treatment, and other factors that we did not measure.

The principal strength of our study is validation of the Duke-PH scale and subscales, showing that their scores predicted both morbidity and utilization while controlling for the effects of 11 other health-related variables.

Limitations of our study are the inclusion of patients in only one community clinic and the use of a convenience sample instead of a random sample. Although the study population was not a random sample, it compared partly with the Federal Uniform Data System (UDS) Lincoln population data (23). For example, although the 73% of study patients who met the federal poverty level was similar to the 72% reported by the UDS, the 29% proportion of Hispanic study patients was much lower than the 47% from the UDS. However, we anticipate that this study population will be similar enough to other diverse community health center populations to provide meaningful comparisons.

Other limitations include the selection of instrument items by the investigators based on their past experience and the incomplete measurement of morbidity and utilization. Morbidity based upon the number of items in the problem list is limited because medical record problem lists may be incomplete, and because simply the presence of a health problem may not be as meaningful a measure of morbidity as the degree of control of that problem. Our assessment of health care utilization would have been better if we had included a variety of follow-up times and weighted follow-up encounters by intensity and resource use. Also, we did not measure usefulness of the instrument.

In summary, we have designed and partly supported reliability, validity, and feasibility of the 14-item Duke-PH, a new brief, self-report, and scorable instrument which is unique because it measures both social and health determinants of population health from the perspective of individuals in the population. Future studies are needed to establish further support for reliability and validity, to compare data in populations of different types, to incorporate more complete measures of morbidity and utilization, and to assess usefulness for improving the well-being of patients.

## Data Availability

The datasets generated for this study are available on request to the corresponding author.

## Author Contributions

GP initiated the study, participated in data collection and analysis, and wrote most of the manuscript. HE made the study possible because of his leadership role as medical director at the study site, assisted significantly in data collection, and writing the manuscript. CC assisted in selection and interpretation of statistical analyses, and in reviewing and revising the manuscript.

### Conflict of Interest Statement

The authors declare that the research was conducted in the absence of any commercial or financial relationships that could be construed as a potential conflict of interest.
